# Loss of intraepidermal nerve fiber density during SIV peripheral neuropathy is mediated by monocyte activation and elevated monocyte chemotactic proteins

**DOI:** 10.1186/s12974-015-0456-8

**Published:** 2015-12-18

**Authors:** Jessica R. Lakritz, Jake A. Robinson, Michael J. Polydefkis, Andrew D. Miller, Tricia H. Burdo

**Affiliations:** Department of Biology, Boston College, Chestnut Hill, MA 02467 USA; Department of Neurology, Johns Hopkins, Baltimore, MD 21231 USA; Department of Biomedical Sciences, Section of Anatomic Pathology, Cornell University College of Veterinary Medicine, Ithaca, NY 14853 USA

**Keywords:** HIV peripheral neuropathy, Intraepidermal nerve fiber density, Monocytes, Dorsal root ganglia

## Abstract

**Background:**

Peripheral neuropathy (PN) continues to be a major complication of human immunodeficiency virus (HIV) infection despite successful anti-retroviral therapy. Human HIV-PN can be recapitulated in a CD8-depleted, simian immunodeficiency virus (SIV)-infected rhesus macaque animal model, characterized by a loss of intraepidermal nerve fiber density (IENFD) and damage to the dorsal root ganglia (DRG). Increased monocyte traffic to the DRG has previously been associated with severe DRG pathology, as well as a loss in IENFD. Here, we sought to characterize the molecular signals associated with monocyte activation and trafficking to the DRGs.

**Methods:**

Eleven SIV-infected CD8-depleted rhesus macaques were compared to four uninfected control animals. sCD14, sCD163, sCD137, regulated on activation normal T cell expressed and secreted (RANTES), and monocyte chemoattractant protein 1 (MCP-1) were measured in plasma and the latter three proteins were also quantified in DRG tissue lysates. All SIV-infected animals received serial skin biopsies to measure IENFD loss as well as BrdU inoculations to measure monocyte turnover during the course of infection. The number of BrdU+ and CD14+ CD16+ peripheral blood monocytes was determined by flow cytometry. The number of MAC387+, CCR2+, CCR5+, and CD137+ cells in DRG tissue was quantified by immunohistochemistry.

**Results:**

sCD14, sCD163, MCP-1, and sCD137 increased significantly in plasma from pre-infection to necropsy. Plasma sCD163 and RANTES inversely correlated with IENFD. Additionally, sCD137 in DRG tissue lysate was elevated with severe DRG pathology and associated with the recruitment of MAC387+ cells to DRG. Elevated numbers of CCR5+ and CCR2+ satellite cells in the DRG were found, suggesting a chemotactic role of their ligands, RANTES, and MCP-1 in recruiting monocytes to the tissue.

**Conclusions:**

We characterized the role of systemic (plasma) and tissue-specific (DRG) monocyte activation and associated cytokines in the pathogenesis of SIV-PN. We identified sCD163 and RANTES as potential biomarkers for HIV-PN, as these were associated with a loss of IENFD. Additionally, we identified CD137 signaling to play a role in MAC387+ cell traffic to DRG and possibly contribute to severe pathology. These studies highlight the role of monocyte activation and traffic in the pathogenesis of SIV-PN, while identifying specific signaling proteins for future pharmacological blockade.

## Background

Human immunodeficiency virus peripheral neuropathy (HIV-PN) continues to be problematic among the HIV-infected population despite successful use of anti-retroviral therapy (ART) to reduce plasma viral loads and increase patient longevity [1]. Distal sensory polyneuropathy (DSP), a common type of HIV-PN, consists of damage to the dorsal root ganglia (DRG) that is associated with loss of distal axons including small diameter unmyelinated nerve fibers that project to the skin and are measured by intraepidermal nerve fiber density (IENFD) [2–4]. Axonal loss and damage to the DRG is thought to occur either via direct neurotoxicity of viral proteins or through indirect mechanisms from chronic immune activation. Application of HIV viral proteins, Tat, gp120, and Vpr in vitro has resulted in neurotoxicity of DRG neurons [5–7]. However, neuronal damage can occur in humans with undetectable viral loads [1], and viral expression in the DRG does not correlate with pathology severity [8]. Instead, in the SIV rhesus macaque animal model, we found that monocyte traffic to the DRG is associated with DRG pathology, as well as a loss of IENFD [8]. However, the underlying mechanism of neurodegeneration in vivo has yet to be fully elucidated.

Monocyte activation is a hallmark of HIV comorbidities among patients on ART [9–11]. Monocyte and macrophage activation, often measured by plasma biomarkers such as sCD14 and sCD163, has been associated with an increase risk of cardiovascular disease, HIV-associated neurocognitive disorders (HAND), renal disease, and frailty [12–15]. Additionally, a greater rate of monocyte egress from the bone marrow is associated with faster progression to AIDS in a CD8-depleted SIV-infected rhesus macaque model of HIV [16]. Furthermore, the CD14+ CD16+ monocyte population expands during HIV infection [17–19], and this expansion of activated monocytes is associated with HAND [18, 20].

Cytokines have a large impact on the nervous system, in addition to regulating the immune response. DRG neurons express cytokine receptors on their surfaces, so they can appropriately respond to cytokines in their environment [21, 22]. CCR5, the receptor for RANTES (Regulated on Activation, Normal T cell Expressed and Secreted)/CCL5, can be expressed on DRG neuronal cell bodies. gp120 can bind to CCR5 resulting in neuronal excitation [23]. CCR2, another chemokine receptor expressed on DRG neurons, can also facilitate neuronal excitation when it binds to monocyte-chemoattractant-1 (MCP-1/CCL2) [24]. Various rodent models of neuropathic pain have demonstrated that blocking the MCP-1-CCR2 interaction via neutralizing antibodies or gene knockout can block pain sensation [25–29]. Neuronal cell bodies in the DRG can also upregulate inflammatory cytokines following peripheral nerve injury [30–32]. Thus, cytokines can transmit pain signals from the periphery to the central nervous system (CNS) via interactions with cytokine receptors on DRG neurons [28, 33]. Additionally, MCP-1 and RANTES might increase recruitment of monocytes to the DRG causing further neuronal damage and activation [21, 34, 35].

Other signaling pathways besides CCR2 and CCR5 are likely involved in neuronal damage during SIV-DSP. One potential signaling protein of interest is CD137, which is a member of TNF superfamily that can be expressed on T cells, monocytes, and other immune cells, as well as endothelial cells [36, 37]. CD137 cross-linking on monocytes induces activation and production of pro-inflammatory cytokines [38]. CD137 expression on endothelial cells facilitates migration of monocytes out of blood vessels and into tissues [37, 39]. Additionally, CD137 reverse signaling is involved in myelopoiesis [40, 41]. Elevated sCD137 in plasma, a splice variant of CD137, has been associated with several inflammation-linked diseases [42–44], but its role in monocyte activation during HIV infection has not been studied.

We used a CD8-depleted, SIV-infected macaque model to recapitulate HIV-DSP in humans, where animals show a loss of IENFD and DRG pathology [45–47]. We have previously demonstrated an influx of activated MAC387+ macrophages to the DRG as well as an increase in CD163+ macrophages. Importantly, we found that increased cell traffic was associated with severe DRG pathology and a greater loss of IENFD [8]. This study sought to investigate the role of monocyte activation in HIV-DSP, as well as identify cytokines that are associated with monocyte activation and neuronal loss in plasma and in DRG tissue.

## Methods

### Ethical statement

All animals used in this study were handled in strict accordance with American Association for Accreditation of Laboratory Animal Care with the approval of the Institutional Animal Care and Use Committee of Harvard University and the Institutional Animal Care and Use Committee of Tulane University.

### Animals, viral infection, and CD8 lymphocyte depletion

Fifteen rhesus macaques (*Macaca mulatta*) were utilized in this study. Eleven animals were inoculated intravenously with SIVmac251 (a generous gift from Dr. Ronald Desrosiers, University of Miami). Four uninfected rhesus macaques served as uninfected controls. All infected animals were administered 10 mg/kg of anti-CD8 antibody subcutaneously at day 6 after infection and 5 mg/kg intravenously at days 8 and 12 after infection in order to achieve rapid progression to AIDS. The human anti-CD8 antibody was provided by the NIH Non-human Primate Reagent Resource (RR016001, AI040101). SIV-infected animals were sacrificed at the onset of terminal AIDS. The development of simian AIDS was determined post-mortem by the presence of *Pneumocystis carinii*-associated interstitial pneumonia, *Mycobacterium avium*-associated granulomatous enteritis, hepatitis, lymphadenitis, and/or adenovirus infection of surface enterocytes in both small and large intestine. Animals were housed at either the New England Primate Research Center (NEPRC; Southborough, MA) or Tulane University’s National Primate Research Center (TNPRC; Covington, LA) in strict accordance with standards of the American Association for Accreditation of Laboratory Animal Care.

### Necropsy and histopathology

Animals were necropsied immediately following death, and representative sections of all major organs were collected, fixed in 10 % neutral-buffered formalin (NBF), embedded in paraffin, and sectioned at 5 μm. After deparaffinization in xylene, the tissues were hydrated in graded alcohols, counterstained with Harris hematoxylin solution (Sigma-Aldrich) for two minutes, and rinsed with running water. The slides were then dipped sequentially in acid alcohol (90 % methanol, 5 % sulfuric acid, 5 % acetic acid; Sigma-Aldrich) and ammonia water (15–20 drops ammonium hydroxide in 250 ml water; Sigma-Aldrich), rinsing with running water after each, followed by 80 % alcohol for 2 min and eosin (Sigma-Aldrich) for 2 min. Tissue sections were then rinsed in graded alcohols and dehydrated with xylene and mounted with VectaMount (Vector).

### Histopathologic analysis of DRG morphology

H and Estained sections of DRG were evaluated blindly for histopathologic lesions by a board-certified veterinary anatomic pathologist (ADM) and scored based on the presence and severity of infiltrating mononuclear cells, neuronophagia, and Nageotte nodules as previously described [8, 46]. Overall pathology was scored on a previously validated [8] scale of 1–3 at increments of 0.5 via the following criteria: (1) Mild: scattered infiltrating mononuclear cells with rare evidence of neuronophagia and/or neuronal loss, (2) Moderate: increased numbers of infiltrating mononuclear cells with occasional neuronophagia and/or neuronal loss, and (3) Severe: abundant infiltrating mononuclear cells, frequent neuronophagia and neuronal loss were all present [8, 46, 48].

### Immunohistochemistry

DRG sections were deparaffinized with xylene and hydrated in a series of graded alcohols. Sections were stained with antibodies against MAC387 (clone M0747; Dako), CCR5 (rabbit polyclonal; Novus Biologicals) or CCR2 (clone 7A7; Abcam). Frozen DRG sections were used for CD137 staining (clone BBK-2). Sections were counterstained with hematoxylin, dehydrated, and mounted using VectaMount permanent mounting medium (Vector Labs). Tissues were visualized using a Zeiss Axio Imager M1 microscope (Carl Zeiss MicroImaging). Quantification of the absolute number and percent of positive satellite cells were performed as previously described [8]. For each animal, eight non-overlapping fields of view at ×200 magnification were quantified by manually counting the number of positive cells in the field and dividing by the total area of DRG tissue. The average number of positive cells per square millimeter was used.

### Skin punch and IENFD measurement

Skin punch biopsy specimens with IENF were performed in all SIV+ animals. Skin punches (3 mm) were taken serially near the sural innervation site just distal to the lateral malleolus. Biopsy specimens were taken for each animal at pre-infection, several time points during infection, and at necropsy. Biopsy specimens were fixed in Zamboni’s fixative and processed for dividing into sections. Sections (50-μm thick) of serial punch skin biopsy specimens were stained with anti-PGP 9.5, a panaxonal marker (1:10,000 dilution; ABD Serotec). Nerve fiber length/volume of epidermis (IENFD) was quantified using computer software (Space balls program; Microbrightfield Bioscience) as previously described [8, 49].

### BrdU administration

A 30 mg/mL stock of solution was prepared by adding 5-bromo-2-deoxyuridine (BrdU; Sigma-Aldrich) to 1× phosphate-buffered saline (without Ca^2+^ and Mg^2+^) and heated to 60 °C in a water bath, as previously described [8, 16, 50]. BrdU was administered as a slow bolus i.v. injection at a dose of 60 mg BrdU/kg body weight. BrdU was administered at 8 and 21 days post-infection (DPI) in animals A01-A07. Additionally, animals A04-A11 received BrdU 42, 63 DPI, and 24 h prior to necropsy.

### Flow cytometry

Flow cytometric analyses were performed with 100 μl aliquots of EDTA-coagulated whole blood. Erythrocytes were lysed using ImmunoPrep Reagent System (Beckman Coulter), washed twice with PBS containing 2 % FBS, and then incubated for 15 min at room temperature with fluorochrome-conjugated surface antibodies including anti-HLA-DR-PerCp-Cy5.5 (clone L243), anti-CD16-PE-Cy7 (clone 3G8), anti-CD3-APC (clone SP34-2), CD8-APC (clone RPA-T8), anti-CD20-APC (clone 2H7), and anti-CD14-Pacific blue (clone M5E2). For intracellular staining, cells were fixed and permeabilized with BD Cytofix/Cytoperm™ buffer (BD Biosciences) for 30 min at room temperature. Cells were again washed and incubated with BD Cytoperm Plus™ buffer for 10 min on ice, then washed and incubated with DNase (30 mg) for 1 h at 37 °C, and washed and then stained for intracellular antigen with anti-BrdU-FITC (clone 3D4; BD Biosciences) and anti-Ki-67-PE (clone B56; BD Biosciences) for 20 min at room temperature. Samples were acquired on a BD FACS Aria (BD Biosciences) and analyzed with Tree Star Flow Jo version 9.6. Identification and quantitation of BrdU+ monocytes and CD14+ CD16+ monocytes was performed as previously described [16].

### Preparation of DRG lysate

Frozen lumbar DRG was mechanically homogenized in Tissue Extraction Reagent I (Invitrogen, Waltham, MA) containing 1× protease inhibitor (Sigma-Aldrich). For every 1 g of tissue, 10 mL of lysis buffer was used. Lysate was centrifuged and supernatant containing protein was stored at −80 °C. Protein was quantified using a BCA protein assay kit (Thermo Scientific) according to the manufacturer’s instructions.

### ELISAs

sCD14 and RANTES were quantified in plasma (diluted 1:200 and 1:4; respectively) using ELISA kits (R&D Systems). sCD163 was quantified in plasma (diluted 1:500) using an ELISA kit (Trillium Diagnostics). All ELISAs were carried out according to the manufacturer’s instructions and as previously described [16].

### Luminex multiplex assays

RANTES, MCP-1, and sCD137 were quantified in DRG tissue lysates, and MCP-1 and sCD137 were quantified in plasma using Multiplex Luminex Technology (EMD Millipore). Non-human Primate Cytokine/Chemokine Panels 1 and 2 were used according to the manufacturer’s instructions with the following modifications. For DRG tissue lysate protein quantification, 10 μg of protein (in 25 μl of lysis buffer) from each sample was loaded onto the plate. Tissue lysis buffer was used as the matrix for dilution of standards and quality control samples. For the plasma sample analysis, plasma samples were diluted two-fold in the assay buffer provided. The provided serum matrix was used for dilution of standards and quality control samples. All samples were performed in duplicate, and plates were incubated overnight at 4 °C on a rocker. Samples were analyzed using MAGPIX System (EMD Millipore).

### Statistical analysis

All statistical analysis was performed using Prism Software (Version 5.0d). A Wilcoxon matched-pairs signed-rank test was used to determine the increase in markers from pre-infection to necropsy. A Mann-Whitney *U* test was used to detect variation between infected and uninfected samples. ANOVA was used to detect variance among different pathology groups followed by a Dunn’s post-test if the ANOVA was significant. Non-parametric Spearman correlation was used for all correlations. A *p* value of <0.05 was considered significant for all tests performed.

## Results

### Animals used for the study

**Fig. 1:**
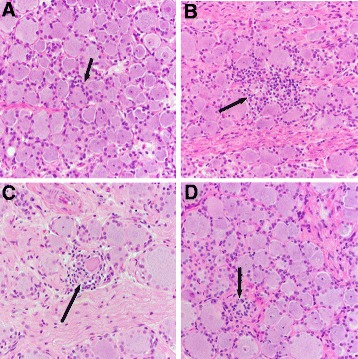
Dorsal root ganglia pathology. **a** DRG with mild focal satellitosis, which is defined as increased numbers of cells around a neuronal cell body (*arrow*). **b** DRG with moderate inflammation that has replaced a few ganglion cells (*arrow*) with an increase in satellite cells (**c**) DRG with inflammation associated with a degenerate ganglion cell (*arrow*). Note the cells encroaching around the outside of the ganglion indicative of neuronophagia, a precursor lesion to the development of a Nageotte nodule. **d** DRG with a Nageotte nodule, focal proliferation of satellite cells that completely replace foci of neuronal cell loss (*arrow*)

Eleven rhesus macaques were infected with SIVmac251 and were administered with a CD8-depletion antibody 6, 8, and 12 DPI in order to rapidly progress to AIDS. All SIV-infected animals developed mild to severe lumbar DRG pathology, as well as a loss of IENFD (Table 1, Fig. 1).

**Table 1:** Animals used in the study

Animal treatment	Animal ID	Survival (days)	% loss of IENFD at necropsy from pre-infection (%)	Lumbar DRG pathology
SIV-infected CD8-depleted	A01	84	−43.3	Severe (3)
	A02	96	−13.0	Mild (1)
	A03	106	−74.5	Moderate (2)
	A04	89	−36.8	Moderate–severe (2.5)
	A05	55	−43.1	Mild–moderate (1.5)
	A06	174	−82.5	Severe (3)
	A07	146	−51.5	Severe (3)
	A08	77	−57.4	Moderate–severe (2.5)
	A09	77	−18.4	Moderate (2)
	A10	168	−20.4	Mild (1)
	A11	97	−51.6^a^	Mild (1)

*IENFD* intraepidermal nerve fiber density

^a^Percent change from pre-infection to 63 DPI

### Plasma markers of monocyte egress and activation during SIV infection

**Fig. 2:**
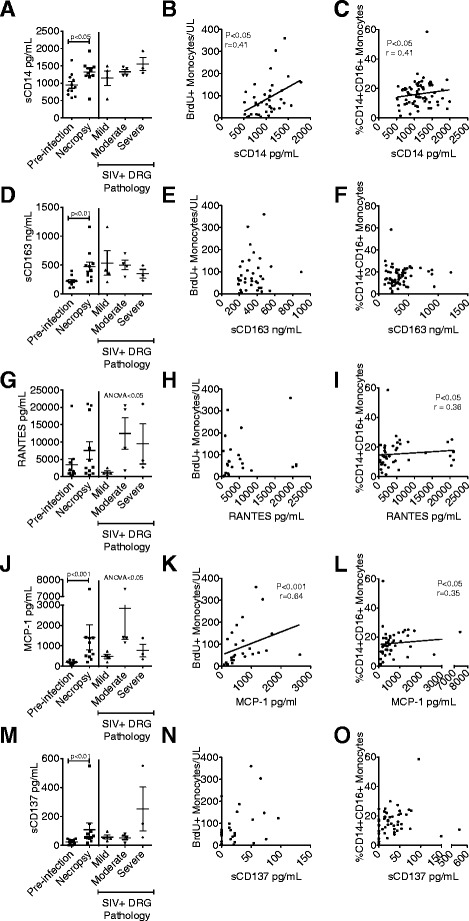
Correlates of plasma markers of monocyte activation and monocyte chemoattractants. sCD14 (**a**–**c**), sCD163 (**d**–**f**), RANTES (**g**–**i**), MCP-1 (**j**–**l**), and sCD137 (**m**–**o**) were measured in plasma at multiple time points throughout infection. **a**, **d**, **g**, **j**, and **m** Pre-infection and necropsy plasma concentrations were compared using a Wilcoxon matched-pairs signed-rank test. Necropsy plasma concentrations were grouped according to the lumbar DRG pathology and compared using a Kruskal-Wallis test. The number of BrdU+ monocytes in blood (**b**, **e**, **h**, **k**, **n**) and the percent of CD14+ CD16+ monocytes of the total monocyte population (**c**, **f**, **i**, **l**, **o**) was determined by flow cytometry and correlated to the plasma soluble protein concentrations at matched time points. A Spearman correlation test was used for all correlations. *p* value of <0.05 was considered significant

Monocyte egress from the bone marrow was measured by BrdU pulse labeling [16]. We also measured CD14+ CD16+ monocytes by multicolor flow cytometry and found that this population of activated monocytes was expanded during SIV infection (data not shown). We investigated soluble monocyte activation markers sCD14 (Fig. 2a–c) and sCD163 (Fig. 2d–f) in plasma and their correlation to the rate of peripheral monocyte turnover and the number of CD14+ CD16+activated peripheral monocytes. Both of these markers increased significantly in our animals from pre-infection to necropsy using Wilcoxon matched-pairs signed-rank test (sCD14 *p* < 0.05; sCD163 *p* < 0.01). sCD14 was also associated with the number of BrdU+ monocytes (Fig. 2b, *p* < 0.05) and the percent of CD14+ CD16+ monocytes out of the total number of circulating monocytes (Fig. 2c, *p* < 0.05). sCD163 did not significantly correlate to the absolute number of BrdU+ or CD14+ CD16+ monocytes (Fig. 2e, f), in contrast to previously published data where percentages and not absolute numbers were examined [16].

Next, we examined the role of RANTES (Fig. 2g–i), MCP-1 (Fig. 2j–l), and sCD137 (Fig. 2m–o) in monocyte egress and activation. We identified sCD137 as a novel signaling protein that may also play an important role in SIV-DSP pathogenesis because of its role in myelopoiesis, monocyte extravasation, and monocyte activation [37–41]. When we compared pre-infection plasma concentrations of RANTES, MCP-1, and sCD137 to necropsy plasma concentrations using the Wilcoxon matched-pairs signed-rank test, we found that MCP-1 and sCD137 both increased significantly during infection (MCP-1, *p* < 0.001; sCD137, *p* < 0.01). RANTES was not increased significantly during infection, but it correlated with the percent of activated CD14+ CD16+ monocytes in circulation at matched time points (*p* < 0.05, Fig. 2i). Additionally, necropsy plasma concentrations of RANTES and MCP-1 were associated with more severe DRG pathology (*p* < 0.05, Fig. 2g, j). MCP-1 was also associated with monocyte egress from the bone marrow (*p* < 0.001, Fig. 2k) and with CD14+ CD16+ monocytes (*p* < 0.05, Fig. 2l). sCD137 did not significantly correlate with either BrdU+ or CD14+ CD16+ blood monocytes (Fig. 2n, o). These data suggest that the rate of monocyte activation and egress from the bone marrow is likely controlled by several soluble factors. Because of this complexity and likely redundancy of pathways, suppressing elevated rates of myelopoiesis is unlikely to be a successful pharmacologic target. In addition, we found that three out of five of our examined proteins to be correlated with the percent of CD14+ CD16+ monocytes, which are typically considered to be the most activated monocyte population, but it is unclear if this population of monocytes is activated by these proteins or producing these signaling factors.

### Correlates of reduced IENFD

**Fig. 3:**
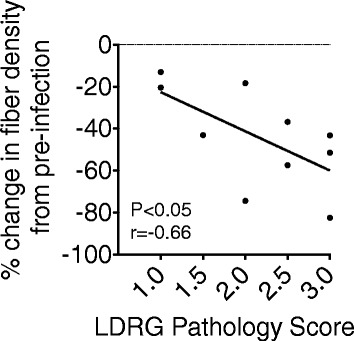
Severe DRG pathology was correlated to a greater loss of IENFD. Lumbar DRG (LDRG) pathology was scored on a scale of 1.0 (mild) to 3.0 (severe). IENFD was measured at pre-infection and necropsy, and the percent change was calculated by dividing the difference in IENFD from pre-infection to necropsy by the pre-infection value and multiplying by 100. Percent change of IENFD was correlated to LDRG pathology using a Spearman correlation test. *p* < 0.05, *r* = −0.66

All 11 SIV-infected animals used in this study had a decrease in IENFD at necropsy, compared to pre-infection, as well as varying degrees of DRG pathology ranging from mild (score of 1) to severe (score of 3) (Table 1). A greater percent loss of IENFD from pre-infection to necropsy was associated with a higher DRG pathology score (Fig. 3; *p* < 0.05). These data show that pathology of DRG and loss of IENFD are linked in severity.

**Fig. 4:**
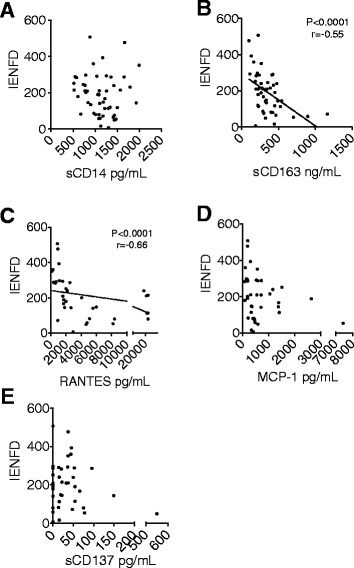
sCD163 and RANTES in plasma negatively correlated to IENFD. IENFD was measured at multiple time points throughout infection in all SIV+ animals. sCD14 (**a**), sCD163 (**b**), RANTES (**c**), MCP-1 (**d**), and sCD137 (**e**) in plasma were correlated to IENFD at matched time points using a Spearman correlation test. *p* value of <0.05 was considered significant

We hypothesized that monocyte activation and chemokines responsible for monocyte traffic to the DRG may facilitate neurodegeneration resulting in a reduced density of nerve fibers in the periphery. To test this, we correlated sCD14 (Fig. 4a), sCD163 (Fig. 4b), RANTES (Fig. 4c), MCP-1 (Fig. 4d), and sCD137 (Fig. 4e) with absolute IENFD at matched time points. sCD163 (*p* < 0.0001) and RANTES (*p* < 0.0001) in plasma negatively correlated with absolute IENFD values. No significant correlation was found for sCD14, MCP-1, and sCD137. Thus, increased monocyte activation and possibly chemotaxis are involved in the dying-back of axons during SIV infection.

### Elevated monocyte chemoattractants in DRG tissue during SIV infection

**Fig. 5:**
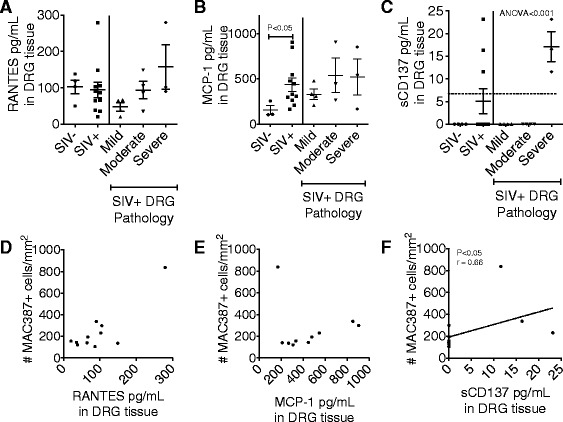
RANTES, MCP-1, and sCD137 protein levels in DRG tissue. RANTES (**a**), MCP-1 (**b**), and sCD137 (**c**) were detected by Luminex multiplex assay in DRG tissue lysate. Differences in protein concentrations in SIV– and SIV+ DRG tissue were analyzed using a Mann-Whitney test. SIV+ DRGs were grouped according to the tissue pathology. Differences between SIV+ tissue pathology groups were analyzed using a Kruskal-Wallis test, followed by a Dunn’s post-test. The amount of RANTES (**d**), MCP-1 (**e**), and sCD137 (**f**) in DRG tissue lysate were correlated to the number of MAC387+ cells/mm^2^ determined by immunohistochemistry using a Spearman correlation test. *p* value of <0.05 was considered significant

Next, we sought to investigate which chemokines are associated with monocyte traffic to the DRG. We have previously shown that an influx of mononuclear cells to the DRG during SIV infection is associated with severe tissue pathology [8]. Thus, we hypothesized that monocyte chemoattractants will be elevated in DRG tissue. Whole DRG tissue, consisting of neurons, satellite cells, and vasculature, were homogenized, and proteins were extracted and analyzed by multiplex assay. There was no detectable difference in RANTES between uninfected and infected DRG tissues, but there was a trend for elevated RANTES in SIV+ DRG with more severe pathology, although this did not reach statistical significance (Fig. 5a). MCP-1 was elevated in SIV+ DRG compared to uninfected control tissue (Fig. 5b; *p* < 0.05). sCD137 was only above the detection level in the three DRG examined with severe pathology (Fig. 5c; ANOVA < 0.001).

To determine if the levels of these chemoattractants in DRGs correlated with the absolute number of MAC387+ recently recruited monocytes in DRGs, we correlated the protein concentrations of RANTES, MCP-1, and sCD137 with the number of MAC387+ monocytes in tissue determined by immunohistochemistry. No significant correlation was found for RANTES or MCP-1 for recruitment of MAC387+ cells (Fig. 5d, e). The amount of sCD137 in DRGs positively correlated with the number of MAC387+ cells in matched DRG tissue (Fig. 5f, *p* < 0.05). These results point to a potential role in sCD137 recruiting MAC387+ monocytes to DRG tissue and facilitating severe tissue damage. Alternatively, MAC387+ cells could be releasing sCD137.

**Fig. 6:**
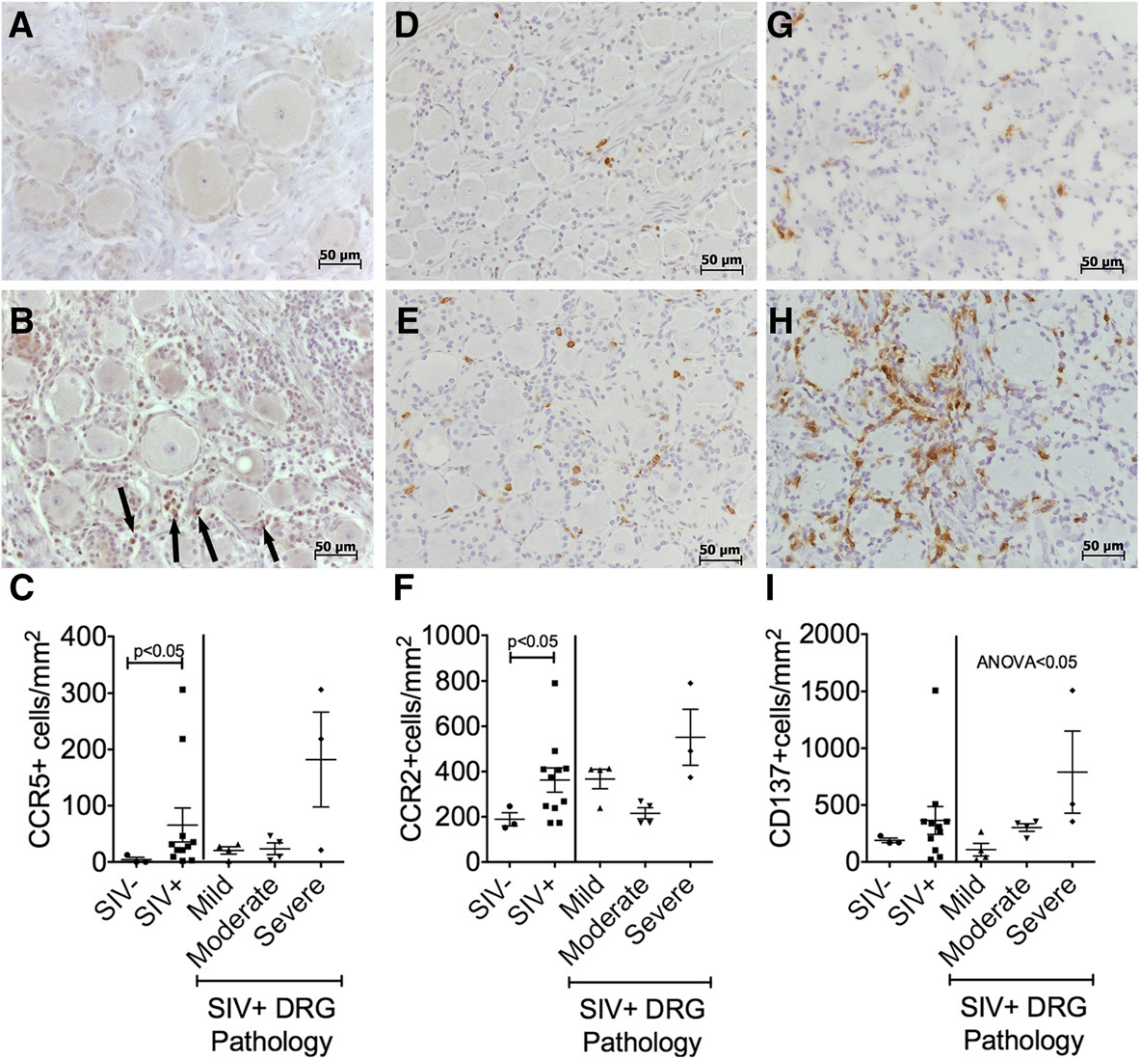
CCR5, CCR2, and CD137 expression on DRG satellite cells. The number of CCR5 (**a**–**c**), CCR2 (**d**–**f**), and CD137 (**g**–**i**) positive satellite cells in DRG tissue was determined by immunohistochemistry in uninfected (**a**, **d**, **g**) and SIV-infected animals (**b**, **e**, **h**). *Arrows* show CCR5+ cells in SIV+ DRG tissue. For each animal, eight non-overlapping fields of view at ×200 magnification were quantified by manually counting the number of positive cells in the field and dividing by the total area of DRG tissue. The average number of positive cells per square millimeter is plotted for each animal (**c**, **f**, **i**). Analysis in SIV− and SIV+ DRG tissue was determined using a Mann-Whitney test. SIV+ DRGs were grouped according to the tissue pathology. Differences between pathology groups determined using a Kruskal-Wallis test, followed by a Dunn’s post-test. *p* value of <0.05 was considered significant

The chemotactic role of RANTES and MCP-1 is well established. MAC387+ cells in the brain of SIV+ macaques are CCR2−. Instead, CCR2 was expressed on perivascular macrophages [51]. Thus, we sought to demonstrate the likely role RANTES and MCP-1 play in recruiting monocytes to DRG. We found increased numbers of CCR5+ (Fig. 6a–c) and CCR2+ (Fig. 6d–f) satellite cells in infected tissue (Fig. 6e, h) compared to uninfected (Fig. 6d, g) controls. The increase of CCR5+ and CCR2+ with SIV infection in DRG was quantitated and found to be statistically significant (Fig. 6c, f; *p* < 0.05 and *p* < 0.05).

sCD137 is generated by alternative splicing [44]. Membrane-bound CD137 is expressed on a wide range of cell types, including monocytes and expression of CD137 facilitates monocyte extravasation into tissue [37, 39, 52]. Thus, we chose to examine membrane-bound CD137 expression on satellite cells in DRGs of SIV− (Fig. 6g) and SIV+ (Fig. 6h) animals. We found that the number of CD137+ cells in SIV+ DRG tissue correlates to tissue pathology (Fig. 6i, ANOVA < 0.05). Because CD137 is not unique to myeloid cells, we performed double immunohistochemistry stains. We found that 16.1 % of CD137+ cells were T cells (CD3+) and 33.5 % of CD137+ cells expressed CD68, a pan-macrophage marker (data not shown). However, because MAC387+ macrophages do not co-express CD68, we suspect the remainder of the CD137+ cells in DRG tissue to be MAC387+ macrophages. However, the MAC387 and CD137 antibodies required different tissue preparation for immunohistochemistry and were incompatible with each other to perform a double stain. Despite this technical pitfall, the correlation of sCD137 to MAC387+ satellite cells in DRG and increased CD137+ satellite cells in DRG with severe pathology highlight a novel potential role of CD137 signaling during SIV infection and DSP pathogenesis.

## Discussion

HIV-PN continues to be a major co-morbidity of HIV infection despite reduction of plasma viral load. Currently, there is no successful treatment for HIV-PN, and thus, understanding the underlying mechanism of neuronal damage is of utmost importance to improve patient quality of life. There is increasing evidence that chronic monocyte/macrophage activation plays a key role in the pathogenesis of HIV/SIV-PN. Animal models of HIV-PN and studies in humans have shown an increase in myeloid cell activation in the DRG and spinal column, as well as a loss of IENFD [3, 8, 53–55]. We have previously demonstrated that increased BrdU+ monocyte traffic to the DRG is associated with severe DRG pathology [8].

Numerous studies have examined the expansion of the CD14+ CD16+ monocyte population during HIV/SIV infection and the link to inflammatory co-morbidities [16, 56, 57]. We have also shown that the rate of monocyte egress from the bone marrow, measured by BrdU pulse labeling, is correlated to faster disease progression to AIDS [16]. To investigate the systemic inflammation that is causing neuronal damage, both in the DRG and in the extremities, we examined five soluble proteins in plasma, which are associated with monocyte activation and traffic. Here, we found that plasma sCD14 and MCP-1 were correlated to the number of BrdU+ monocytes in blood. We also found that sCD14, RANTES, and MCP-1 were correlated to the percent of CD14+ CD16+ monocytes out of total blood monocytes. CD14+ CD16+ monocytes are an activated population of monocytes that highly express CCR2, the receptor for MCP-1, and CD163 [58, 59]. Elevated levels of RANTES and MCP-1 in plasma were associated with moderate or severe DRG pathology, compared to mild pathology. These findings confirm these soluble factors in plasma are associated with monocyte activation and traffic during SIV-PN.

Because the blood-nerve barrier is more promiscuous (or leakier) than the blood-brain barrier [60], we assumed that neurons were exposed to all proteins found in plasma. Several inflammatory cytokines have been found to be neurotoxic in vitro [61, 62], but this direct causation is difficult to prove in vivo*.* We found a significant inverse correlation between sCD163 and IENFD. Thus, sCD163 may be useful as a plasma biomarker of IENFD loss in HIV+ patients. While sCD163 and sCD14 are both markers of monocyte activation, they are shed by different mechanisms. CD163 is highly expressed on M2-polarized macrophages and CD14+ CD16+ monocytes, while CD14 is present on all populations of monocytes and is shed in the setting of non-specific activation [63] and CD163 is shed due to cell-surface TLR activation [64]. Plasma RANTES/CCL5 also correlated to a reduction in IENFD. Other studies have demonstrated that the supernatant of macrophages exposed to gp120, presumably containing proteins such as sCD163 and RANTES, is capable of damaging neurons in vitro [61]. Even though sCD163 and RANTES strongly correlated to a reduction of IENFD, the dying back of axons is likely caused by several signals.

Here, we found a link between the severity of DRG pathology and a greater loss of small nerve fiber density in the footpad. It is unknown if a loss of IENFD comes before damage to the DRG or vice versa. One study suggested that damage to the DRG preceded altered functional activity of nerve fibers in the periphery [65]. However, we have observed an early loss of IENFD (as early as 8 DPI) and minimal DRG pathology in animals sacrificed at 21 DPI (data not shown). Regardless, we found an association between pathology at the DRG and in the footpad, suggesting a relay of signals from one region to the other, or perhaps systemic neuroinflammatory/neurotoxic proteins that facilitate damage to both regions simultaneously.

To investigate the local signals in the DRG responsible for monocyte traffic, we analyzed DRG tissue homogenate using a multiplex assay that allowed for quantification of many proteins with a small amount of tissue homogenate available. The limitation of this method is that it is unknown which cell types are producing the proteins that were detected. Endothelial cells, neurons, Schwann cells, and immune cells (including macrophages and T cells) in the DRG are all capable of secreting cytokines and chemokines. However, this method still affords us the opportunity to investigate the local signals responsible for monocyte traffic and neuronal damage at the DRG. We found that MCP-1 in DRG tissue was significantly increased in DRG from SIV+ animals compared to uninfected control tissue. Because MCP-1 is a potent monocyte chemoattractant and is produced by activated macrophages, it is likely that MCP-1 is partially responsible for increased monocyte traffic to the DRG.

In addition to the proteins we reported on in detail here, we also investigated other known monocyte chemoattractants, both in the DRG and in plasma. We did not find a significant increase in MIP-1α, MIP-1β, and MIP-3α in plasma nor were these proteins elevated in SIV+ DRG tissue lysate. In fact, these proteins were below detection level for many of the DRG samples tested. However, monocyte activation and traffic is a complex process, likely to be controlled by several signaling molecules that were not included on the two cytokine/chemokine panels we utilized.

sCD137/CD137 (formally called 4-1BB or tumor necrosis factor receptor superfamily member 9 (TNFRSF9)) has not been extensively studied in the context of monocyte activation during HIV infection, although its known functions in other diseases are relevant to HIV pathogenesis. Here, CD137 was found to potentially play a role in SIV-DSP pathogenesis. The soluble form of CD137 (sCD137) is generated by alternative splicing and was found to significantly increase in plasma from pre-infection to terminal AIDS. Additionally, sCD137 was only detectable in DRG lysate with severe pathology, and it correlated with the number of MAC387+ cells in DRG tissue. CD137 is expressed on a wide range of cell types, although most of the research on this protein focuses on T cell activation [36–38]. However, CD137 signaling has been shown to play a role in myelopoiesis, monocyte activation, and monocyte extravasation into inflamed tissue [37, 38, 41]. The known roles of CD137 in regard to monocyte activity are also highly deregulated during HIV/SIV infection. Additional research needs to be conducted in order to further define the role of CD137 signaling in HIV/SIV disease progression. CD137 activation, through the use of agnostic monoclonal antibodies, has proven to have potential for cancer treatment by stimulating the immune system to target cancer cells [66]. Blocking CD137 may ameliorate chronic immune activation seen during HIV/SIV infection.

## Conclusions

Our findings presented here demonstrate the complexity of the neuro-immune interaction that occurs during the pathogenesis of SIV-DSP. Neurons are capable of producing cytokines and express cytokine receptors. Stimulation through these receptors has been shown to modulate pain signaling [33, 62]. However, targeting a single cytokine or a receptor is likely not to reverse or prevent nerve damage due to redundancy of immune signaling. No single protein that was investigated in this study was found to associate with all the factors we know to be important in nerve damage during SIV infection. However, sCD163 and RANTES were identified as potential biomarkers for loss of IENFD. Additionally, elevated sCD137 in DRG tissue lysate was found to be associated with MAC387+ cell recruitment and severe pathology. The role of CD137 signaling during SIV-PN pathogenesis warrants further investigation. Future studies should focus on blockade of multiple signals which may dampen monocyte activation and traffic to the DRG and thus prevent a loss of IENFD and DRG damage.

## Abbreviations

ART: anti-retroviral therapy
BrdU: bromodeoxyuridine
DPI: days post-infection
DRG: dorsal root ganglia
DSP: distal sensory polyneuropathy
HAND: HIV-associated neurocognitive disorders
HIV: human immunodeficiency virus
IENFD: intraepidermal nerve fiber density
MCP-1: monocyte chemoattractant protein 1
PN: peripheral neuropathy
RANTES: regulated on activation, normal T cell expressed and secreted
SIV: simian immunodeficiency virus
